# Enhanced thermal properties of novel shape-stabilized PEG composite phase change materials with radial mesoporous silica sphere for thermal energy storage

**DOI:** 10.1038/srep12964

**Published:** 2015-08-11

**Authors:** Xin Min, Minghao Fang, Zhaohui Huang, Yan’gai Liu, Yaoting Huang, Ruilong Wen, Tingting Qian, Xiaowen Wu

**Affiliations:** 1 Beijing Key Laboratory of Materials Utilization of Nonmetallic Minerals and Solid Wastes, National Laboratory of Mineral Materials, School of Materials Science and Technology, China University of Geosciences (Beijing), Beijing 100083, P.R. China; 2 School of Chemical Engineering, University of Birmingham, Edgbaston, Birmingham B15 2TT, UK

## Abstract

Radial mesoporous silica (RMS) sphere was tailor-made for further applications in producing shape-stabilized composite phase change materials (ss-CPCMs) through a facile self-assembly process using CTAB as the main template and TEOS as SiO_2_ precursor. Novel ss-CPCMs composed of polyethylene glycol (PEG) and RMS were prepared through vacuum impregnating method. Various techniques were employed to characterize the structural and thermal properties of the ss-CPCMs. The DSC results indicated that the PEG/RMS ss-CPCM was a promising candidate for building thermal energy storage applications due to its large latent heat, suitable phase change temperature, good thermal reliability, as well as the excellent chemical compatibility and thermal stability. Importantly, the possible formation mechanisms of both RMS sphere and PEG/RMS composite have also been proposed. The results also indicated that the properties of the PEG/RMS ss-CPCMs are influenced by the adsorption limitation of the PEG molecule from RMS sphere with mesoporous structure and the effect of RMS, as the impurities, on the perfect crystallization of PEG.

Industry is the driving engine of economic development, and energy must be seen as the fuel for this engine. However, both the shortage of non-renewable energy resources and the increasingly serious environmental problems caused by the use of fossil fuels restrict the healthy development of social economy. Thus, increasing attention on the developing and utilizing new green energy sources has been attracted worldwide. Worth mentioning in these resources is a technique of latent heat storage employing phase change materials (PCMs), which have become a hotspot in the study of thermal energy storage materials due to their high energy storage density, isothermal operating characteristics, and extremely small temperature variation during charging and discharging processes^1^,^2^,^3^. Today, PCMs have been widely used in many fields such as building energy conservation, solar heating system, thermal regulating textiles, temperature-control greenhouses and air-conditioning systems^4^,^5^,^6^.

Based on the chemical composition, PCMs can be grouped into two categories, namely organic and inorganic ones^1^. Compared to inorganic PCMs, such as salt hydrates and their mixtures, organic PCMs possess numerous advantages of low-price, small corrosion, having no phenomenon of phase separation and undercooling^7^,^8^,^9^. Among various organic PCMs, polyethylene glycol (PEG) is the most promising one due to its suitable phase change temperature and high latent heat storage capacity, which can be tuned through varying molecular weight. Besides, PEG also owns congruent melting behavior, good chemical and thermal stabilities, excellent corrosion resistance, non-toxicity, biodegradation, low vapor pressure, and competitive price^10^,^11^,^12^. However, the actual applications of PEG inevitably face three enduring problems: phase instability in melting state, low thermal conductivity, and weak interfacial combination with the supporting materials, which limit its further applications^10^,^13^,^14^.

In order to solve these problems, a novel shape-stabilized composite phase change material (ss-CPCM) composed of PEG and inorganic supporting material was developed^12^,^15^. Comparing with the organic ones, inorganic materials have a better chemical and thermal stability, thermal conductivity, and flame retardancy, indicating that inorganic supporting matrix can be used as promising PCMs with enhanced thermal properties^16^,^17^,^18^,^19^. Moreover, the powder–like organic–inorganic ss-CPCMs can always keep solid state even when the solid phase-change material turned to liquid. During the phase-change process, the supporting materials can make liquid PCMs easy to handle and protect PCMs from harmful interactions with the surrounding materials and environment^20^,^21^,^22^.

Among various inorganic supporting materials, porous materials are attractive, but less studied in the phase change systems. If porous materials are used, the phase change properties and shape stabilization of PCMs were directly related to the average pore size of the porous materials^23^. In detail, if the average pore size is too small, the PCM molecular motion will be impeded. On the contrary, there will not be sufficient capillary force to stabilize the liquid PCMs. Ref. [24] reported that a mesoporous support material could perform better than others. Thus, PEG based composite PCMs stabilized by mesoporous matrices are promising candidates for high performance heat storage systems. Among them, mesoporous silica, as an important inorganic amorphous material has stimulated great interest due to their superior properties, such as low density, large BET specific areas, unique pore structures, great surface permeability, high adsorption capacity, desirable thermal conductivity, non-photodegradable inorganic framework, ecofriendly nature, and fire resistant^23^,^24^,^25^,^26^, indicating that silica can be considered as a nice supporting material.

Recently, synthesis of mesoporous silica with special morphology has stimulated great research interest due to their superior properties^27^,^28^,^29^,^30^,^31^. Therefore, these functional materials have proven to be promising in widespread applications, including sorption/separation catalysis, drug delivery/release, lithiumion batteries, sensors^32^,^33^. Among them, it is worth mentioning that the fibrous structured silica nanospheres, because of their Radial direct channels and large pore size, help target molecules reach the adsorption sites more easily than other mesoporous materials^34^,^35^. However, to the best of our knowledge, this kind material used as supporting material has never been reported.

In this paper, a new PEG/SiO_2_ ss-CPCM based on radial mesoporous silica (RMS) with enhanced thermal properties for thermal energy storage was prepared via vacuum impregnation operation. The radial mesoporous silica (RMS) carriers were first synthesized via a facile self-assembly process with CTAB as the main template and TEOS as SiO_2_ precursor. The resulting PEG/SiO_2_ ss-CPCM will be a potential candidate for the application in the fields of building envelopes in the continuous hot summer, whose temperature is often as high as 50 ~ 70 °C.

## Results

### Formation, morphology, structure and surface properties of RMSs

**Figure 1:**
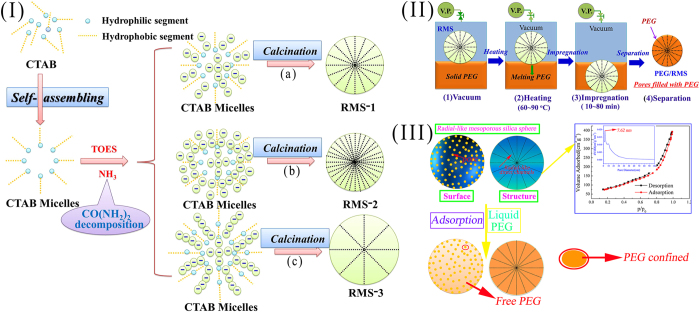
(I) The schematic formation processes of RMS; (II) The vacuum impregnation treatment for preparing phase change composites (VP: vacuum pump); (III) Schematic formation mechanism of PEG/RMS composite.

The proposed mechanism of the formation of RMS spheres is shown in Fig. 1(I). The microstructure of templates stemming from CTAB is one key for the texture properties of the resultant mesoporous materials^36^. Firstly, the radial template is quickly formulated by the spontaneous self-assembly of CTAB micelles^34^,^35^. Simultaneously, the hydrolysis of TEOS occurred in cyclohexane with water and OH^−^ (produced by urea decomposition), producing a large amount of negative charges silicate molecules. Since CTAB is a kind of cationic surfactant, it is naturally deduced that it is easily connected with the silicate molecules through electrostatic attractions along the free radial directions and the restricted tangential direction. Finally, condensation of the self-assembled silicate in the available spaces among the Radial CTAB micelles leads to the crystallization of the silica material. The templates could be removed after the calcination of the materials at 650 °C. Thus, the RMS was obtained. Moreover, when the concentration of TEOS is certainly increased, the ordered silica fibers might thicken from Fig. 1-I(b) (RMS-2). Decreasing the amount of TEOS would cause a decline of silicate molecules, and the aggregation number of CTAB micelles is increased, resulting in the enlargement of the particle size (RMS-3), which is well consistent with literatures^34^,^35^.

**Figure 2:**
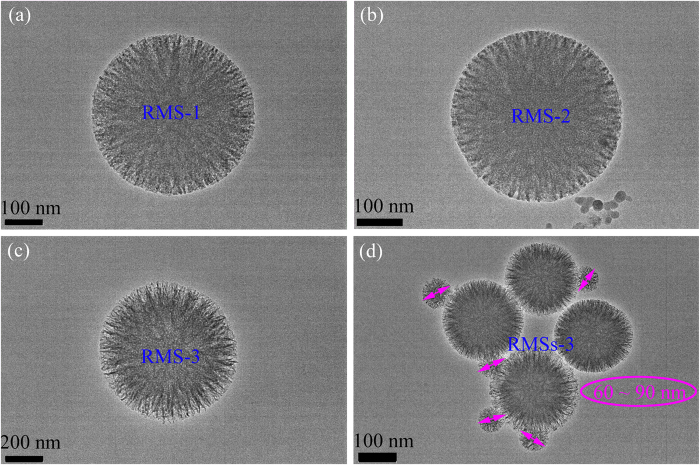
TEM images of (**a**) RMS1, (**b**) RMS2, and (**c**,**d**) RMS3.

The detailed structural and morphological features of the samples were further examined by TEM (Fig. 2). All the samples possess an interesting radial morphology with fibers coming out from the center and distributed uniformly in all directions. Notably, when other conditions were kept constant during the synthesis, an increase in the amount of TEOS had almost no influence on the length of the fibrous silica shell but made the layer much denser, which proves the formation of RMSs in Fig. 1-I(b). Besides, when the molar ratios of TEOS/CTAB and other conditions were kept constant during the synthesis, more CTAB would be available to form larger micelles, resulting in the enlargement of the particle size and sparseness of the silica layer, which also agrees with the formation mechanism of RMS-3 in Fig. 1-I(c). Interestingly, in Fig. 2(d), fibrous silica particles with the diameter of only 60 ~ 90 nm were also found, which was not reported before. Finally, RMS-1 was selected as the supporting material for preparation of PEG/RMS ss-CPCM.

### Chemical compatibility analysis of the prepared PEG/RMS ss-CPCM

**Figure 3:**
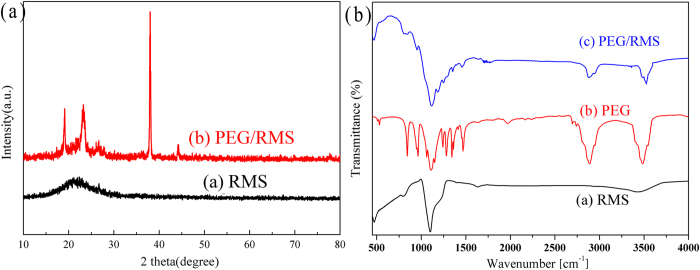
(**a**) XRD patterns of PEG and the prepared PEG/RMS ss-CPCM (**b**) FT-IR spectrums of PEG, RMS and PEG/RMS ss-CPCM.

Figure 1-II presents the preparation of PEG/RMS phase change composites by the vacuum impregnation treatment. And the chemical compatibility between PEG and SiO_2_ was determined via XRD and FT-IR analysis. Figure 3(a) shows the XRD pattern of the prepared RMS and PEG/RMS ss-CPCM. In Fig. 3(a), the broad peak between 20 ~ 30° indicates a typical non-crystalline structure of RMS. The peaks around 19°, 23°, 38° and 43° are assigned to PEG crystal, indicating that the crystal structure of PEG is not destroyed. Figure 3(b) shows the FTIR spectra of PEG, RMS and PEG/SiO_2_ ss-CPCM. From Curve (1), the peak at 1094 cm^−1^ corresponds to the asymmetric stretching vibration of Si-O-Si and the bands at 796 and 486 cm^−1^ can be assigned to the symmetric stretching and deformation modes of Si-O-Si. All of these characteristic peaks belong to SiO_2_. From Curve (2), the typical stretching vibrations of C–H at 953 and 2887 cm^−1^ correspond to –CH_2_ of PEG. And the stretching vibrations of C–O and –OH are found at 1107 and 3487 cm^−1^, respectively. From Curve (3), all the main absorption peaks of both PEG and SiO_2_ appear as predicted except for some slight peak shifts and no obvious new peak is observed, indicating that there is only physical absorption between PEG and SiO_2_ matrix other than chemical interaction. These findings further verify that no chemical interaction occurs between PEG and SiO_2_. And the physical combination between PEG and SiO_2_ can maintain the stable thermal performance of PEG and avoid the influence of chemical forces.

### Micro-morphology analysis of the prepared PEG/RMS ss-CPCM

**Figure 4:**
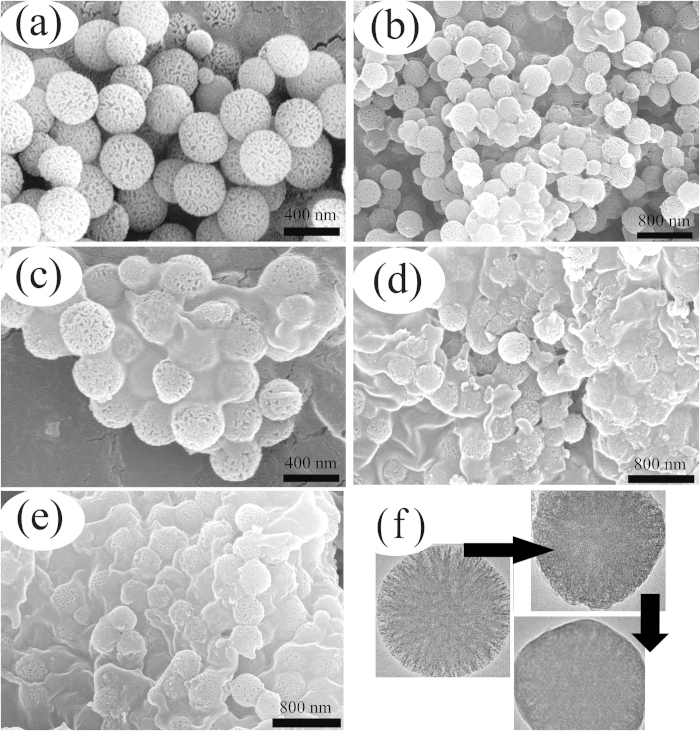
SEM pictures of RMS (**a**) and ss-CPCMs with various PEG mass fractions (wt.%, **b**: 50; **c**: 60; **d**: 70; **e**: 80); (**f**) TEM images of PEG/RMS ss-CPCM with different PEG contents (60 wt.% and 80 wt.%).

Figure 4 presents the microstructures of RMS and ss-CPCMs prepared with different PEG mass fractions. It can be seen that RMS is mainly composed of monodisperse spherical particles with fibrous morphology, and these particles are non-aggregated with narrow size distribution, indicating the high porosity and large specific surface area of RMS as expected. The average diameter of RMS is calculated to be about 400 nm (Fig. 4a). From Fig. 4(b–e), the PEG with different mass ratios (50%, 60%, 70% and 80%) in the composites was adsorbed and dispersed in the porous network of the RMS, respectively. The PEG was distributed well in the composites due to the effect of capillary and surface tension forces between the PEG and the porous network of the active RMS. Figure 4(f) shows the TEM image of RMS particle and PEG/RMS particles with 60% and 80% PEG. There are still some empty pores for 60%PEG/RMS, while in 80%PEG/RMS composite, nearly all pores are evenly filled by PEG and the microstructure shows good physical compatibility compared to the structure of RMS. The RMS carrier material provided the good mechanical strength for the whole composite and prevented the seepage of the melted PEG. Therefore, the shape-stabilized CPCMs were obtained.

The chemical and textural properties of RMS and PEG/RMS ss-CPCMs with different PEG weight percentages are summarized in Table 1. From Table 1, we observe that the BET surface areas, pore volumes and diameters of the PEG/RMS composite decreased with the increase in PEG weight percentages and were far lower than those of RMS. The results proved the analysis from SEM pictures, indicating that PEG is uniformly distributed into RMS pores.

### Phase change behavior of the prepared PEG/RMS ss-CPCM

**Figure 5:**
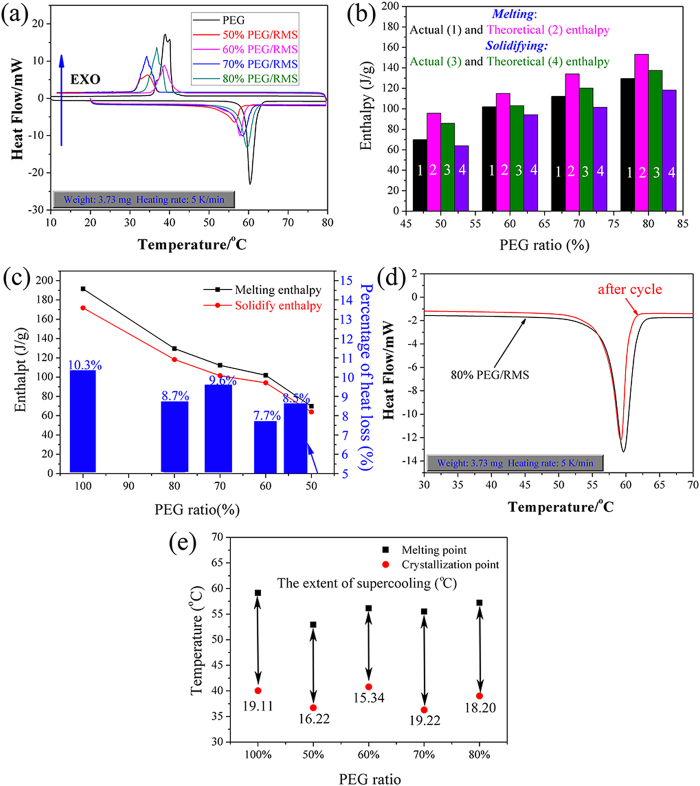
PEG and PEG/RMS ss-CPCMs. (**a**) DSC curves; (**b**) Comparison of theoretical and actual enthalpies; (**c**) Percentage of heat loss. (**d**) Melting curves of 80% PEG/RMS before and after thermal cycle experiment. (**e**) Phase change temperatures of pristine PEG and the prepared 80% PEG/SiO_2_ ss-CPCM.

Phase changing behavior is described with two parameters, thermal energy storage capacity and phase changing temperature, which can be determined by means of DSC technique. Figure 5(a) demonstrates the melting and solidifying DSC curves of PEG and PEG/RMS composite. The phase change parameters obtained from DSC evaluation are summarized in Table 2. According to the literature^14^, the theoretical enthalpy of ss-CPCM could be determined by Eq. (1):


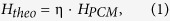


where *H*_*theo*_ is the theoretical enthalpy of the ss-CPCM; η is the mass fraction of the PEG; *H*_*PCM*_ represents the actual latent heat of the pristine PEG PCM. However, from Fig. 5(b), we observed that the actual enthalpies of ss-CPCMs are much lower than their theoretical values. Similar phenomenon has been obtained by Feng *et al*^23^. It was likely to be caused by the interference of mesoporous matrices with the crystallization of PEG: the drag and steric effect restricted the crystal arrangement and orientation of PEG molecular chains, resulting in the decline of regularities of crystal line regions and the increase of lattice defects; therefore, the actual enthalpies of ss-PCMs were lower than theoretical enthalpies.

Additionally, it was observed that the melting enthalpy is larger than the solidifying vaule of PEG/RMS composite. This may due to fact that mass loss increases when the sample is heated from 25 to 100 °C during melting process test by the DSC. Thereafter, the solidifying process test of MEPCM is carried out. Because of mass loss of PEG/RMS in the melting process test, the solidifying latent heat of MEPCM is smaller than the melting latent heat of MEPCM in the solidifying process test. The heat storage efficiency (γ) is evaluated using the heat loss percentage by Eq. (2):


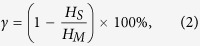


The comparisons of phase change enthalpy and heat loss percentage for pure PEG and the composite PCMs are shown in Fig. 5(c). Compared with pure PEG, the melting and solidifying enthalpies of the composite PCMs declined with increase in RMS content. This is because the blending of mesoporous matrices decreases the weight percentage of PEG and interferes with the crystallization of PEG. Therefore, 80%PEG/RMS could be unquestionably chosen as the most promising latent heat storage material. From Fig. 5(c), the heat loss percentage of pure PEG between endothermic and exothermic cycles was higher than that of the composite PCMs.

In order to determine the reliability of 80%PEG/RMS, the 200-cycle experiment was conducted and no leakage of PEG was found. As shown in Fig. 5(d), no significant difference can be observed in the endothermal curve after cycles, indicating that 80%PEG/RMS has excellent thermal reliability, which means a longer life cycle.

As shown in Table 2, the melting temperature of the prepared PEG/RMS composite was lower than that of pristine PEG, which may be caused by the physical interactions and confined effect of PEG and RMS, or the enhancement in thermal conductivity^37^. Qian *et al.*^13^ and Wang *et al.*^38^ once pointed out that the weak interactions between PCMs and porous materials, such as surface tension, capillary forces, and hydrogen bonding, would cause a decline in phase change temperature. Moreover, the addition of inorganic silica enhanced the thermal conductivity of ss-CPCMs, which resulted in a rapid temperature response; therefore, the melting temperature reduced.

Moreover, as an important parameter in practical applications, supercooling of PCMs must be considered. Based on the DSC measurement results from Table 2, the extent of supercoiling could be evaluated as the difference between the melting and solidifying temperature. The evaluation results are shown in Fig. 5(e). Compared with that of pristine PEG, the extent of supercooling of the prepared PEG/RMS ss-CPCM is reduced by 4.8% ~ 19.7%. This suggests that the extent of supercooling of PEG can be favorably reduced by blending with mesoporous RMS.

### Effects of impregnation time and temperature

**Figure 6:**
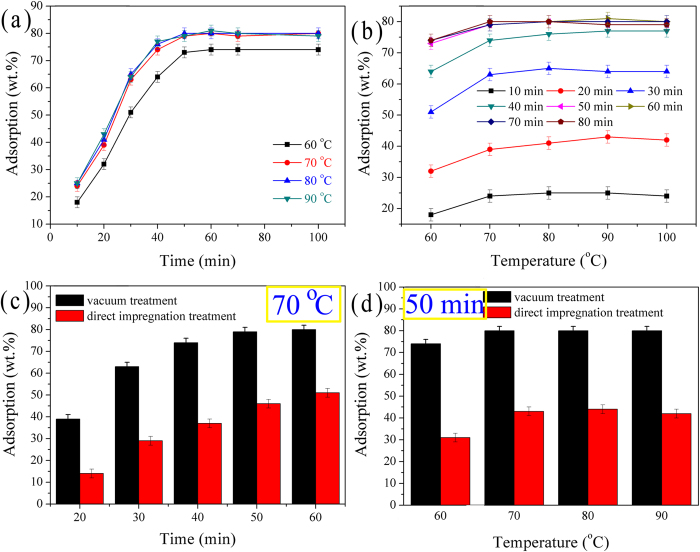
(**a**,**b**) Effects of impregnation time and temperature on PEG adsorption capacity. (**c**,**d**) Comparison between vacuum and direct impregnation method.

Figure 6(a,b) demonstrate the effects of impregnation time and temperature on the PEG adsorption capacity. In Fig. 6(a), the adsorption capacity increases rapidly in the initial stage. After 50 min, the adsorption capacity remains almost unchanged. In Fig. 6(b), PEG adsorption capacity increases significantly when the temperature rises to 70 °C and remains nearly unchanged when the temperature is above 70 °C. After 50 min, the immersion temperature has a slight effect on the adsorption capacity. Since the increases of immersion time and temperature were not effective, the immersion time of 50 min and the immersion temperature of 70 °C were regarded as the optimal conditions for the preparation of PEG/RMS ss-CPCM.

### Comparison with natural immersion

Based on the literatures^15^,^16^, those ss-CPCMs prepared by the vacuum method usually have the better adsorption capacity and thermal property. For some references^12^,^13^, however, there has been no distinction between the two. Therefore, the two methods were compared (Fig. 6(c,d)). The obtained values are the average values of at least three samples with the relative error less than 3%. As shown in the figures, the difference between the absorption results of the vacuum impregnation and natural immersion is quite clear. There is more than twice as much increment in the absorption of vacuum impregnation method comparing to that of natural immersion. Possible reason may be found in their pore structures. The liquid PEG can be adsorbed into pores due to capillary forces, but the atmosphere pressure within the pores prevent the impregnation. And the operation will turn out to be a failure if the capillary forces are smaller than the atmospheric pressure. It is more difficult for liquid PEG to permeate into pore space with smaller diameter than that with larger diameter. So the vacuum impregnation method is more effective and necessary for the porous materials with finer pores. Moreover, those composite PCMs in which pores are not fully filled with PEG could not be used as shape-stabilized CPCMs because air within the pores will expand dramatically at the elevated temperature and cause the leakage of the PEG.

### Thermal stability of the prepared PEG/RMS ss-CPCM

**Figure 7:**
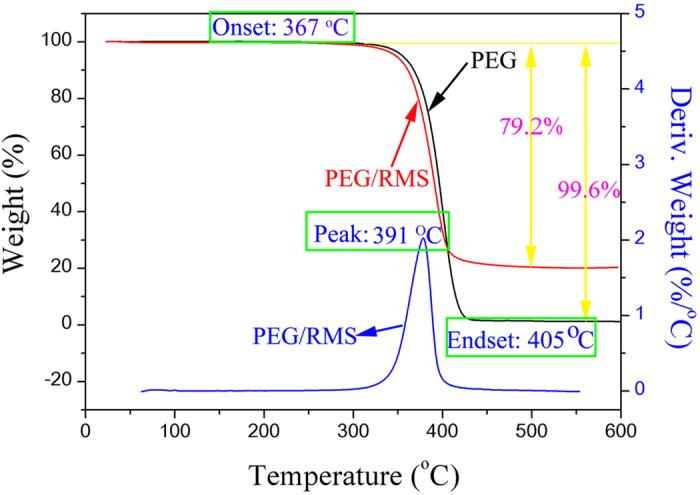
TGA curves of pristine PEG and PEG/RMS ss-CPCM and the corresponding DTG thermograms of PEG/RMS ss-CPCM.

Thermal stability, which plays a significant role in thermal energy storage applications, is one critical parameter for ss-CPCMs. Figure 7 presents the TGA and DTG thermograms of PEG and the prepared PEG/RMS ss-CPCM. As shown in Fig. 7, the weight loss processes of both pure PEG and ss-CPCM were carried out by only one step. As for ss-CPCM, no apparent decomposition reaction and weight loss were found from 25 °C to 367 °C. Therefore, the ss-CPCM has good thermal stability when the temperature is below 367 °C. This property is needed for a material to be used in heat storage applications. However, PEG components in the composites began to decompose as the temperature exceeded 367 °C, and basically stopped after 405 °C. Corresponding to the degradation process, the sharp weight loss within the temperature around 390 °C is ascribed to the decomposition of organic ingredients, namely the breaking of the PEG chains. Moreover, only 0.4 wt.% unknown residues were remained for PEG and the residual mass of PEG/RMS ss-CPCM was 19.8 wt.%, suggesting that the prepared PEG/RMS ss-CPCM was homogeneous. And the loading content (79.2 wt.%) of PEG in the PCM composite from the TGA measurements is in good agreement with the value obtained in the experiment.

## Discussion

Figure 1-III illustrates the schematic formation mechanism of PEG/RMS ss-CPCM. PEG segments could be adsorbed by RMS through capillary force and surface tension. Nevertheless, RMS will more or less interfere with the solidifying process of PEG molecules. Figure 1-III also shows the N_2_ adsorption-desorption isotherm and pore size distribution curve of the RMS sample. In the isotherms, RMS resembled a typical type IV isotherm with a hysteresis loop of type H1 in the P/P_0_ range of 0.4–1.0, indicating the mesoporous nature of RMS. The main pore size is located about 7.62 nm; some nano-sized pores exited as well. Since some of the PEG chains were inevitably embedded into the nano-sized pores, while other PEG chains were adsorbed on the surface of the mesoporous matrices. The combined effects of nano-sized and mesoporous confinement and surface adsorption impeded the crystal aggregation of the whole PEG chain, which has been proved by the DSC results. Though the abundant pores of RMS restrict PEG’s free movement, they can also stabilize the PEG/RMS ss-CPCMs above the melting point of PEG. These results are good in agreement with that of reference^39^.

## Methods

### Materials

PEG, as the latent heat storage material, was in commercial grade with an average molecular weight of 4000 and purchased from Beijing Chemical Reagent Ltd. All other chemical reagents (TEOS, CTAB, n-pentanol, cyclohexane, urea, etc.) used were analytically pure and purchased from Beijing Chemical Reagent Ltd. Deionized water was used throughout the experiments.

### Preparation of RMSs

The RMSs are synthesized from emulsion system using CTAB as surfactants and TEOS as a source of silica. In a typical synthesis, TEOS was added to 20 mL of cyclohexane and 1 mL of n-pentanol to form solution A. CTAB was added in an aqueous solution (20 mL) containing urea (0.4 g) to form solution B. Solution B was quickly added to solution A under constant stirring. Then the mixture was transferred to a Teflon-lined autoclave and heated at 130 °C for 4 h. After cooling to the room temperature, the resultant product was separated centrifugally for 3 times, washed with water, dried in a vacuum oven at 60 °C for 12 h to release water molecules adsorbed in the pores of the SiO_2_. Finally, after the materials were calcined at 650 °C for 3 h, RMS was obtained.

### Preparation of PEG/RMS ss-CPCM

PEG/RMS ss-CPCMs were prepared using the vacuum impregnation operation and the corresponding vacuum impregnation device used in literature [15] has been applied in this paper. The RMS particles were first placed inside a filtering flask, which was connected to water tromp apparatus to evacuate air from its porous surface. Then, liquid PEG was allowed to flow into the flask to cover RMS sample. After the vacuum process was to be continued for heated for 10 ~ 80 min at 60 ~ 90 °C with the vacuum pressure of 65 kPa, air was allowed to enter the flask again to force the liquid PEG to penetrate into the pore space of the RMS. Following this procedure the PEG was impregnated with different mass fractions (50 ~ 80 wt.%). The mixture was cooled and grounded. Finally, PEG/RMS shape-stabilized composite phase change materials were obtained.

### Analysis methods

The specific surface area and pore volume of RMSs were determined by a N_2_ adsorption analyzer (Quantachrome Instruments, US). Transmission electron microscope (TEM, JEM-2100HR, Japan) and scanning electronic microscope (SEM, Model HITACHI S-4800) was adopted to observe the microstructures of ss-CPCMs. The chemical compatibility of CPCMs was obtained via Fourier transform infrared spectroscopy (FT-IR, Model Frontier) and X-ray diffraction (XRD, Model XD-3) method. Besides, thermal property and stability of the CPCMs were explored through differential scanning calorimeter (DSC, Q2000) and thermo-gravimetric analysis (TGA, Q50), respectively.

## Additional Information

**How to cite this article**: Min, X. *et al.* Enhanced thermal properties of novel shape-stabilized PEG composite phase change materials with radial mesoporous silica sphere for thermal energy storage. *Sci. Rep.* **5**, 12964; doi: 10.1038/srep12964 (2015).

## Figures and Tables

**Table 1:** Chemical and textural properties of RMS and PEG/RMS ss-CPCMs.

Sample	PEG (wt.%)	BET (m^2^·g^−1^)	Pore volume (cm^3^·g^−1^)	Mean Pore Diameter (nm)
RMS-1	0.0	284.7	0.543	7.62
RMS-2	0.0	189.2	0.267	7.15
RMS-3	0.0	297.3	0.576	7.43
50%TiO_2_/RMS	50	127.4	0.214	2.41
60%TiO_2_/RMS	60	45.7	0.136	0.89
70%TiO_2_/RMS	70	9.13	0.082	0.37
80%TiO_2_/RMS	80	2.51	0.006	0.05

**Table 2:** Thermal characteristics of PEG and the prepared PEG/RMS ss-CPCMs.

PEG contents	Melting Process		Solidifying Process	
	H_M_ (J/g)	T_M_ (^o^C)	H_S_ (J/g)	T_S_ (^o^C)
100%	191.50	59.15	171.80	40.04
50%	69.85	52.92	63.92	36.70
60%	102.0	56.13	94.15	40.79
70%	112.3	55.50	101.5	36.28
80%	129.6	57.22	118.3	39.02
